# The efficacy of azithromycin to prevent exacerbation of non-cystic fibrosis bronchiectasis: a meta-analysis of randomized controlled studies

**DOI:** 10.1186/s13019-022-01882-y

**Published:** 2022-10-11

**Authors:** Kui Li, Li Liu, Yan Ou

**Affiliations:** Department of Respiratory and Critical Care Medicine, Chongqing Bishan District People’s Hospital, 57 Canghou Lane, Bishan, Chongqing, 400000 China

**Keywords:** Azithromycin, Non-cystic fibrosis bronchiectasis, Exacerbation, Randomized controlled trials

## Abstract

**Introduction:**

The efficacy of azithromycin to prevent exacerbation for non-cystic fibrosis bronchiectasis remains controversial. We conduct this meta-analysis to explore the influence of azithromycin versus placebo for the treatment of non-cystic fibrosis bronchiectasis.

**Methods:**

We have searched PubMed, EMbase, Web of science, EBSCO, and Cochrane library databases through July 2019 for randomized controlled trials (RCTs) assessing the efficacy of azithromycin versus placebo for non-cystic fibrosis bronchiectasis. This meta-analysis was performed using the random-effect model.

**Results:**

Four RCTs were included in the meta-analysis. Overall, compared with control group for non-cystic-fibrosis bronchiectasis, azithromycin treatment was associated with improved free of exacerbation (odd ratios [OR] = 3.66; 95% confidence interval [CI] = 1.69–7.93; *P* = 0.001), reduced pulmonary exacerbations (OR = 0.27; 95% CI 0.13–0.59; *P* = 0.001) and number of pulmonary exacerbations (standard mean difference [SMD] =  − 0.87; 95% CI − 1.21 to − 0.54; *P* < 0.00001), but demonstrate no obvious impact on forced expiratory volume in 1 s (FEV1), score on St George’s respiratory questionnaire, nausea or vomiting, adverse events.

**Conclusions:**

Azithromycin is effective to prevent exacerbation of non-cystic fibrosis bronchiectasis.

## Introduction

Bronchiectasis is featured by the inflammation and remodeling of bronchial wall, and is regarded as the end result of multiple diseases [1–3]. The inflammation mechanism is an exaggerated and uncontrolled neutrophilic responses which is triggered by an abnormal activation of pro-inflammatory cytokines such as interleukin-1b, interleukin-8, tumour necrosis factor-a and leukotrienes [4–6]. Blood and exhaled breath condensate levels of hydrogen peroxide, superoxide anion, 8-isoprostane and reactive nitrogen intermediates are elevated in patients with cystic fibrosis, bronchiectasis and chronic obstructive pulmonary disease [7–10].

Many antibiotics are developed for the control of bacterial infection during the exacerbations of bronchiectasis [11–13]. Growing evidences indicated that some drugs provided beneficial effects. For instance, macrolides can reduce the number of exacerbations and the decline in lung function, and improve health-related quality of life and survival rate in patients with bronchiectasis [14–17]. In a double-blind clinical trial, long-term macrolide maintenance therapy is effective to reduce the frequency of exacerbations in patients with chronic obstructive pulmonary disease or bronchiectasis [18–21].

Long-term prophylaxis with azithromycin has been reported to reduce the frequency of exacerbations and sputum volume, and improve lung function values in patients with non-cystic fibrosis bronchiectasis [22, 23]. Several studies have investigated the efficacy and safety of azithromycin for non-cystic fibrosis bronchiectasis, but the results are conflicting [7, 20, 24]. This meta-analysis of RCTs aims to assess the efficacy and safety of azithromycin treatment versus placebo for non-cystic fibrosis bronchiectasis.

## Materials and methods

This meta-analysis was performed based on the guidance of the Preferred Reporting Items for Systematic Reviews and Meta-analysis statement and Cochrane Handbook for Systematic Reviews of Interventions [25, 26]. No ethical approval and patient consent were required because all analyses were based on previous published studies.

### Literature search and selection criteria

We have systematically searched several databases including PubMed, EMbase, Web of science, EBSCO and Cochrane library from inception to July 2019 with the following keywords: “azithromycin” AND “bronchiectasis”. The reference lists of retrieved studies and relevant reviews were also hand-searched and the process above was performed repeatedly in order to include additional eligible studies.

The inclusion criteria were presented as follows: (1) study design was RCT, (2) patients were diagnosed with non-cystic fibrosis bronchiectasis, and (3) intervention treatments were azithromycin versus placebo.

### Data extraction and outcome measures

Some baseline information was extracted from the original studies, and they included first author, number of patients, age, female, body mass index, forced expiratory volume in 1 s (FEV1) and detail methods in two groups. Data were extracted independently by two investigators, and discrepancies were resolved by consensus. The primary outcomes were free of exacerbation and pulmonary exacerbations. Secondary outcomes included number of pulmonary exacerbations, FEV1, score on St George’s respiratory questionnaire, nausea and vomiting, adverse events.

### Quality assessment in individual studies

The methodological quality of each RCT was assessed by the Jadad Scale which consisted of three evaluation elements: randomization (0–2 points), blinding (0–2 points), dropouts and withdrawals (0–1 points) [27]. One point would be allocated to each element if they were conducted and mentioned appropriately in the original article. The score of Jadad Scale varied from 0 to 5 points. An article with Jadad score ≤ 2 was considered to have low quality. The study is thought to have high quality if Jadad score ≥ 3 [28].

### Statistical analysis

We assessed standard mean difference (SMD) with 95% confidence interval (CI) for continuous outcomes (number of pulmonary exacerbations, FEV1, and score on St George’s respiratory questionnaire) and odd ratios (OR) with 95% CI for dichotomous outcomes (free of exacerbation, pulmonary exacerbations, nausea and voimiting, adverse events). Heterogeneity was evaluated using the I^2^ statistic, and I^2^ > 50% indicated significant heterogeneity [29]. The random-effects model was used for all meta-analysis. We searched for potential sources of heterogeneity when encountering significant heterogeneity. Sensitivity analysis was performed to detect the influence of a single study on the overall estimate via omitting one study in turn or performing the subgroup analysis. Owing to the limited number (< 10) of included studies, publication bias was not assessed. Results were considered as statistically significant for P < 0.05. All statistical analyses were performed using Review Manager Version 5.3 (The Cochrane Collaboration, Software Update, Oxford, UK).

## Results

### Literature search, study characteristics and quality assessment

**Fig. 1:**
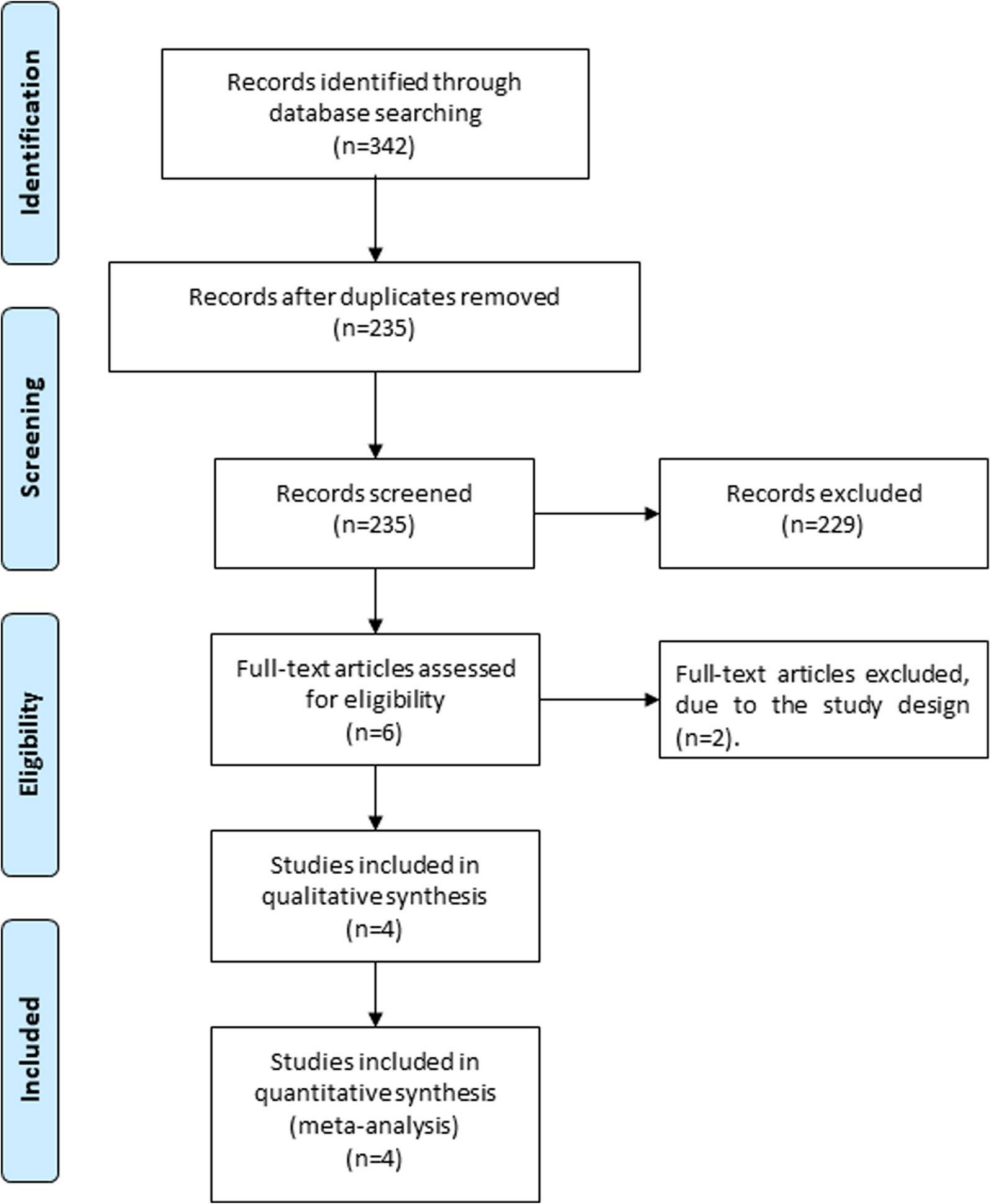
Flow diagram of study searching and selection process

Figure 1 showed the detail flowchart of the search and selection results. 342 potentially relevant articles were initially identified and four RCTs were finally included in the meta-analysis [7, 20, 24, 30].

The baseline characteristics of four included RCTs were shown in Table 1. These studies were published between 2012 and 2013, and the total sample size was 343. Three studies reported adults [7, 20, 24], while one study reported children [30]. The treatment duration ranged from 3 to 24 months, and detail methods of azithromycin differed in each RCT.

**Table 1:** Characteristics of included studies

NO	Author	Azithromycin group						Control group						Jada scores
		Number	Age (years)	Female (n)	Body mass index (kg/m.^2^)	FEV1 (L)	Methods	Number	Age (years)	Female (n)	Body mass index (kg/m.^2^)	FEV1 (L)	Methods	
1	Valery 2013	45	3.99 ± 2.14	19	–	–	azithromycin (30 mg/kg) once a week for up to 24 months	44	4.22 ± 2.30	23	–	–	matched placebo	4
2	Diego 2013	16	57 ± 11	9	27 ± 1	1.48 ± 0.8	Azithromycin 250 mg three times per week during 3 months	14	61 ± 12	7	24 ± 2	1.63 ± 0.7	Matched placebo	4
3	Altenburg 2013	43	59.9 ± 12.3	25	23.0 ± 3.4	–	Azithromycin (250 mg daily) for 12 months	40	64.6 ± 9.1	28	24.5 ± 4.0	–	Matched placebo	5
4	Wong 2012	71	60.9 ± 13.6	48	28.8 ± 7.2	1.87 ± 0.74	500 mg azithromycin three times a week for 6 months	70	59.0 ± 13.3	50	28.6 ± 6.9	1.88 ± 0.69	Matched placebo	5

FEV1, forced expiratory volume in 1 s

Two studies reported free of exacerbation and pulmonary exacerbations [20, 30], three studies reported number of pulmonary exacerbations [7, 20, 30], two studies reported FEV1 and score on St George’s respiratory questionnaire [7, 24], two studies reported nausea and vomiting [24, 30], as well as adverse events [20, 24, 30]. Jadad scores of four included studies varied from 3 to 5, and all four studies had high-quality.

### Primary outcomes: free of exacerbation and pulmonary exacerbations

**Fig. 2:**
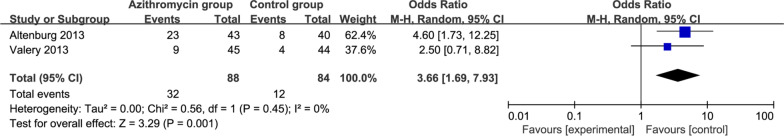
Forest plot for the meta-analysis of free of exacerbation

**Fig. 3:**
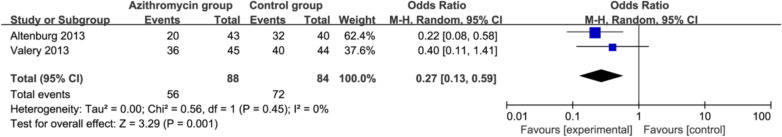
Forest plot for the meta-analysis of pulmonary exacerbations

The random-effect model was used for primary outcomes. The results found that compared to control group for non-cystic-fibrosis bronchiectasis, azithromycin treatment was associated with significantly improved free of exacerbation (OR = 3.66; 95% CI 1.69–7.93; *P* = 0.001) with no heterogeneity among the studies (I^2^ = 0%, heterogeneity *P* = 0.45, Fig. 2) and reduced pulmonary exacerbations (OR = 0.27; 95% CI 0.13–0.59; *P* = 0.001) with no heterogeneity among the studies (I^2^ = 0%, heterogeneity *P* = 0.45, Fig. 3).

### Sensitivity analysis

There was no heterogeneity for primary outcomes, and thus we did not perform sensitivity analysis by omitting one study in each turn to detect the source of heterogeneity.

### Secondary outcomes

**Fig. 4:**
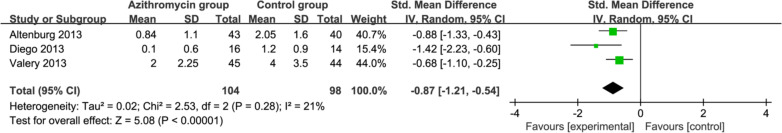
Forest plot for the meta-analysis of number of pulmonary exacerbations

**Fig. 5:**
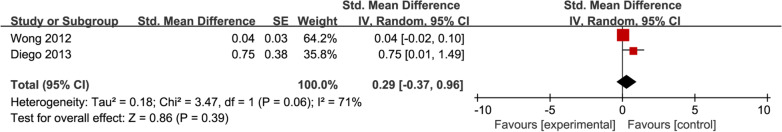
Forest plot for the meta-analysis of FEV1 (L)

**Fig. 6:**
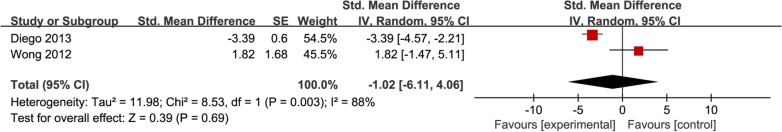
Forest plot for the meta-analysis of score on St George’s respiratory questionnaire

**Fig. 7:**
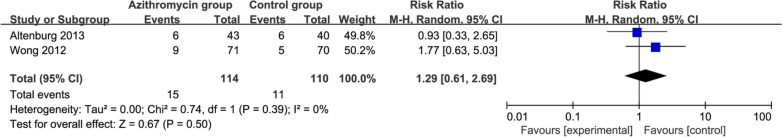
Forest plot for the meta-analysis of nausea and vomiting

**Fig. 8:**
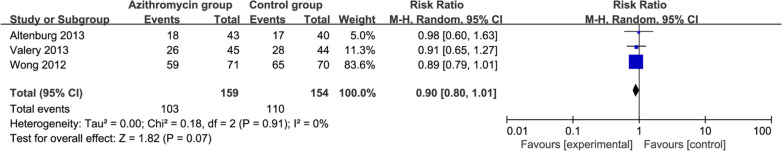
Forest plot for the meta-analysis of adverse events

In comparison with control intervention for non-cystic-fibrosis bronchiectasis, azithromycin treatment can substantially reduce the number of pulmonary exacerbations (SMD =  − 0.87; 95% CI − 1.21 to − 0.54; *P* < 0.00001; Fig. 4), but had no obvious influence on FEV1 (SMD = 0.29; 95% CI − 0.37 to 0.96; *P* = 0.39; Fig. 5), score on St George’s respiratory questionnaire (SMD =  − 1.02; 95% CI − 6.11 to 4.06; *P* = 0.69; Fig. 6), nausea or vomiting (OR = 1.29; 95% CI 0.61–2.69; *P* = 0.50; Fig. 7), or adverse events (OR = 0.90; 95% CI 0.80–1.01; *P* = 0.07; Fig. 8).

## Discussion

Several studies have reported the benefit of azithromycin for non‐cystic fibrosis bronchiectasis [18–21, 24, 31]. In one open-label prospective study of 30 patients, azithromycin at the dose of 250 mg three times per week for 3 months resulted in a significant reduction in sputum volume, symptoms, health-related quality of life and frequency of exacerbations for non‐cystic fibrosis bronchiectasis [7]. Long-term azithromycin treatment was found to provide beneficial effects on exacerbations, forced expiratory volume in 1 s and sputum volume for these patients [22].

Our meta-analysis suggested that azithromycin treatment can substantially improve free of exacerbation, reduce pulmonary exacerbations and number of pulmonary exacerbations for non-cystic-fibrosis bronchiectasis, but showed no significantly favorable impact on FEV1 or St George’s respiratory questionnaire. In the EMBRACE trial, 6 months of azithromycin treatment was found to reduce the rate of event-based exacerbations and increase the time to the first exacerbation compared with placebo [24]. In addition, azithromycin can produce a significant improvement in health-related quality of life and disease symptoms in the BAT trial [20].

In a previous study, azithromycin was reported to provide beneficial effects regardless of infection [16]. In contrast, the post-hoc analysis comparing the effect of treatment in patients with or without P. aeruginosa, the benefits are more pronounced in patients with P. aeruginosa [7]. Macrolides act through the inhibition of cell chemotaxis, cytokine synthesis, adhesion molecule expression and reactive oxygen species generation [7]. No increase in adverse events is observed after azithromycin based on the results of this meta-analysis. In addition, bronchiectasis are divided into nonperfused and perfused types [32]. The surgical indication of bronchiectasis is localized bronchiectasis documented by high-resolution computed tomography and obvious symptom such as chronic productive cough, repeated or significant hemoptysis, recurrent pulmonary infection and failure of medical treatment [33].

Several limitations exist in this meta-analysis. Firstly, our analysis is based on only four RCTs, and more RCTs with large sample size should be conducted to explore this issue. Next, although there is no significant heterogeneity, different duration and methods of azithromycin therapy and characteristics of patients may have some influence on the pooling results. Finally, some unpublished and missing data may lead to some bias for the pooled effect.

## Conclusion

Azithromycin treatment benefits to prevent the exacerbations among patients with non-cystic fibrosis bronchiectasis.

## Data Availability

Not applicable.

## Abbreviations

RCTs: Randomized controlled trials
MDs: Mean differences
CIs: Confidence intervals
RRs: Risk ratios
